# SMARCB1 (INI1) retained but SMARCA4 (BRG1) negative atypical teratoid/rhabdoid tumor arising at the bilateral cerebellopontine angles: a case report

**DOI:** 10.1093/jscr/rjab400

**Published:** 2021-09-30

**Authors:** Nobuyuki Mitsui, Kensuke Oikawa, Mishie Tanino, Manabu Kinoshita

**Affiliations:** Department of Neurosurgery, Asahikawa Medical University, Asahikawa, Japan; Department of Pathology, Asahikawa Medical University, Asahikawa, Japan; Department of Diagnostic Pathology, Asahikawa Medical University Hospital, Asahikawa, Japan; Department of Neurosurgery, Asahikawa Medical University, Asahikawa, Japan

**Keywords:** atypical teratoid/rhabdoid tumor, bilateral cerebellopontine angles, SMARCB1 (INI1), SMARCA4 (BRG1)

## Abstract

Most atypical teratoid/rhabdoid tumor (AT/RT) of the central nervous system shows an inactivation of SMARCB1 (INI1) and is considered as the hallmark of this neoplasm. However, AT/RT could exceptionally rarely present retained SMARCB1 (INI1) but inactivated SMARCA4 (BRG1). Here, the authors report a rare case of a 2-year-old boy with a SMARCB1 (INI1) retained but SMARCA4 (BRG1) negative AT/RT arising at the bilateral cerebellopontine angles mimicking neurofibromatosis type 2. The tumor was highly aggressive and was refractory to all treatment modalities. This case highlights the challenges during differential diagnosis of atypical cerebellopontine angle tumors of childhood and the importance of thoroughly investigating SMARCB1 (INI1) and SMARCA4 (BRG1) when AT/RT is suspected.

## INTRODUCTION

Atypical teratoid/rhabdoid tumor (AT/RT) of the central nervous system (CNS), first described by Rorke *et al.* in 1987 and further defined in a subsequent report in 1996, is an aggressive, highly malignant rhabdoid tumor of early infancy and childhood. This tumor has often been radiologically and histologically confused with medulloblastoma (MB) but has a much worse prognosis. The reported 2-year survival rate of patients with AT/RT is ~15%, which is much lower than the 85%-5-year survival rate of patients with a standard-risk MB [1, 2]. Therefore, discrimination of AT/RT from MB is of clinical importance. Furthermore, the current 2016 WHO classification defines AT/RT as ‘a malignant CNS embryonal tumor composed predominantly of poorly differentiated elements and frequently including rhabdoid cells, with inactivation of SMARCB1 (INI1) or (extremely rarely) SMARCA4 (BRG1)’ [3–5]. We report a case of SMARCB1 (INI1) retained, but SMARCA4 (BRG1) negative highly aggressive AT/RT with unusual neuroimaging presentation of the disease mimicking neurofibromatosis type 2 (NF2), where the disease invaded the bilateral auditory canals.

## CLINICAL SUMMARY

**Figure 1:**
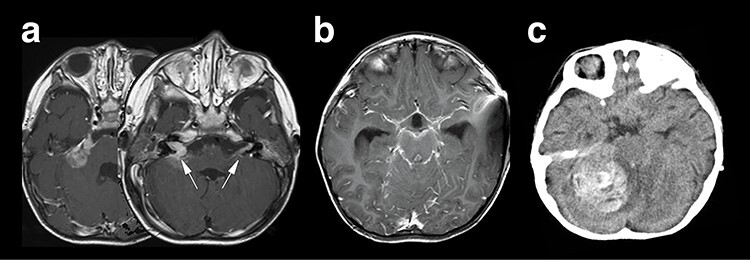
Neuroradiological representation of the lesions, T1WI showed a homogeneously enhanced lesion at the cerebellopontine angle spreading to the bilateral auditory canal (white arrows) (**a**), and a widespread leptomeningeal enhancement was encasing the brainstem (**b**). Unfortunately, the patient’s condition suddenly deteriorated, and the CT image revealed a massive intra-tumoral hemorrhage compressing the brainstem (**c**).

**Figure 2:**
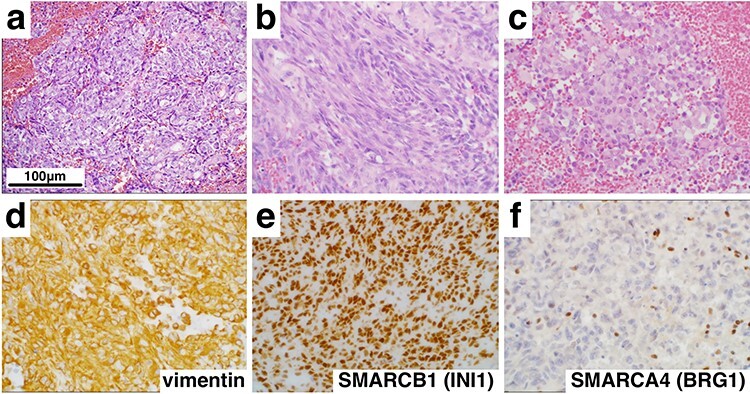
Histological and immunohistochemical findings (×400). Hematoxylin and eosin staining revealed a tumor comprising epithelioid (**a**), spindle cells (**b**) and rhabdoid cells (**c**). In addition, immunohistochemistry showed diffuse positivity for vimentin (**d**) and SMARCB1 (INI1) (**e**), but loss of SMARCA4 (BRG1) (**f**) expression.

Right hemifacial paresis was the chief complaint of this 2-year-old boy. Initial magnetic resonance imaging (MRI) revealed a solitary lesion in the right cerebellopontine (CP) angle extending into the internal auditory canal (Fig. 1a). A contrast-enhanced T1-weighted image (T1WI) revealed another lesion at the contralateral left facial and vestibulocochlear cranial nerve (Fig. 2a arrows). This case was initially considered as NF2 based on bilateral auditory canal invasion of the tumor. His general condition worsened within 2 weeks, with the tumor hemorrhagically expanding and accompanying communicating hydrocephalus. The patient underwent an emergency ventriculoperitoneal shunt placement, which alleviated his condition. Subsequent neuroimaging revealed that widespread leptomeningeal enhancement surrounded the brainstem and the spinal cord and occupied the subdural and subarachnoid space (Fig. 1b). He developed a sudden severe conscious disturbance 4 weeks after the initial onset of the disease with massive intra-tumoral hemorrhage compressing the brainstem (Fig. 1c). An emergency tumor resection was conducted, achieving subtotal removal of the lesion. The patient’s general condition generally deteriorated, and he died 3 months after the operation, although chemotherapy (ifosfamide, cisplatin, etoposide) was prescribed. The overall survival of the patient was 3.5 months.

## PATHOLOGICAL FINDINGS

On histopathologic examination, the tumors contained variable components with epithelial (Fig. 2a) and mesenchymal features (Fig. 2b) accompanied with rhabdoid tumor cells showing eosinophilic inclusions and eccentric nuclei with prominent nucleoli (Fig. 2c). Numerous mitotic figures, apoptotic bodies and focal necrosis were present. Immunohistochemical stains demonstrated elevated MIB-1 (Ki-67) proliferation index of 60%, strong and diffuse expression of vimentin (Fig. 2d) and SALL4, scattered reactivity for keratin, epithelial membrane antigen, glial fibrillary acidic protein, neurofilament (NF), synaptopghysin and S-100. Immunopositivity for SMARCB1 (INI1) was retained (Fig. 2e), but the loss of expression of SMARCA4 (BRG1) in nuclei of tumor cells was confirmed (Fig. 2f). These findings supported a diagnosis of AT/RT, WHO grade IV, with inactivation of SMARCA4 (BRG1).

## DISCUSSION

The CNS AT/RT is a specific entity of pediatric embryonic tumor entity. This tumor type comprises 6.7% of pediatric brain tumors in the age group under 2-year-old and 1.3% of tumors in the age group under 17-year-old [6]. They are often radiographically and histologically confused with MB. According to a large review of AT/RT, 63% of cases had infra-tentorial masses, 27% supratentorial masses, 8% presented with multiple lesions and 2% had tumors within the extramedullary cervical spinal cord [7]. The difference in tumor location between AT/RT and MB is a valuable feature to discriminate between these two diseases. While 80% of classic MB arise in the cerebellum, 47–52% of AT/RT occur in the posterior fossa [8].

On the other hand, it is challenging to distinguish AT/RT from MB based on the radiographical characteristics of MRI [8]. The tumor is more likely to be AT/RT than MB if a posterior fossa tumor in a child shows restricted diffusion involving the CP angle [9]. The CP angle lesion on the right side of the current patient showed restricted diffusion on MRI and could have been suggestive of AT/RT. However, it should be noted that the enhanced lesion on the left side showed non-specific findings.

Since the first report in 1999 describing the mutation of SMARCB1 (INI1) in AT/RT in 1999 [10], detecting loss of INI1 nuclear protein (the product of SMARCB1/INI1 gene) became a convenient diagnostic tool for AT/RT [10–14]. However, up to 15–20% of this tumor showed no alterations in the SMARCB1/INI1 gene and posed a significant diagnostic challenge [10, 12]. Moreover, mRNA of SMARCB1/INI1 was detected in some AT/RT patients despite INI1 proteins being negative on immunohistochemistry [1]. This finding suggested that some unknown post-transcriptional regulatory mechanism of the INI1 protein exists in AT/RT tumor cells. In line with this controversy, several cases of AT/RT were reported with loss of protein expression of the ATPase subunit BRG1 protein (SMARCA4/BRG1 gene) due to a homozygous nonsense mutation in AT/RT [4, 13]. There are only a few cases reported regarding SMARCB1 (INI1) retained, but SMARCA4 (BRG1) negative AT/RT and SMARCA4 (BRG1) negative AT/RT tend to show more aggressiveness compared with SMARCB1 (INI1) retained tumors (average overall survival 3 months versus 24 months) [5, 15]. To the best of our knowledge, the presented case is the first case of this entity resembling NF2. Although the importance of investigating SMARCA4 (BRG1) status in SMARCB1 (INI1) retained suspected AT/RT is advocated today, this unfortunate case highlights the challenges during differential diagnosis of atypical CP angle tumors of childhood, which consist of a variety of rare tumor types.
